# Seroepidemiological evidence of severe fever with thrombocytopenia syndrome virus infections in wild boars in Nagasaki, Japan

**DOI:** 10.1186/s41182-016-0009-6

**Published:** 2016-04-03

**Authors:** Daisuke Hayasaka, Yu Fuxun, Akira Yoshikawa, Guillermo Posadas-Herrera, Satoshi Shimada, Mya Myat Ngwe Tun, Masanobu Agoh, Kouichi Morita

**Affiliations:** Department of Virology, Institute of Tropical Medicine (NEKKEN), Nagasaki University, 1-12-4 Sakamoto, Nagasaki, 852-8523 Japan; Leading Graduate School Program, Nagasaki University, 1-12-4 Sakamoto, Nagasaki, 852-8523 Japan; Nagasaki Prefectural Institute for Environmental Research and Public Health, Omura, Nagasaki, 856-0026 Japan; Present address: National Institute of Infectious Diseases, 1-23-1 Toyama, Shinjuku-ku Tokyo, 162-8640 Japan

## Abstract

Severe fever with thrombocytopenia syndrome (SFTS) is an emerging disease in East Asia. It is thought that the SFTS virus (SFTSV) circulates between ticks and animals in nature and that the virus is transmitted to humans by tick bites. SFTS is endemic to Nagasaki in western Japan; however, epidemiological information regarding SFTSV in Nagasaki is not known. In this study, we performed SFTSV IgG ELISAs and neutralization antibody assays for a seroepidemiological survey using samples from wild boars captured in six areas of Nagasaki. SFTSV seropositive animals were found in three areas. Our findings provide epidemiological information on the distribution of SFTSV in Nagasaki.

## Introduction

Severe fever with thrombocytopenia syndrome (SFTS) is an emerging disease that was first reported in China and has been identified in South Korea and Japan [1–4]. The causative agent, SFTS virus (SFTSV), belongs to the genus *Phlebovirus* in the family *Bunyaviridae* [4]. Humans appear to be infected by the bite of an infected tick, such as *Haemaphysalis longicornis* [5]. Seroepidemiological surveys have demonstrated that anti-SFTSV antibodies have been identified in domestic and wild animals, including sheep, cattle, and dog, in endemic area of SFTS [6–8], indicating that SFTSV circulates between ticks and animals in nature.

The clinical symptoms of SFTS include fever, enteritis, thrombocytopenia, and leukopenia, with fatality rates of up to 30 % [2, 4, 9, 10]. No specific treatment or vaccines for SFTS are currently available. Thus, an epidemiological survey that provides distribution of SFTSV in ticks and animals will be of help for the prevention of the disease in endemic areas.

In Japan, more than 140 cases of SFTS have been identified since 2005 http://kanpoken.pref.yamaguchi.lg.jp/jyoho/page9/sfts_1.php. In Nagasaki located on the Japanese island of Kyushu, seven cases were identified by 2014 [2]. We previously reported that neither virus isolation nor viral gene detection was confirmed in tick pools that included more than 2000 ticks collected in Nagasaki [11]. This indicates that the epidemiological survey of SFTSV in ticks may not provide enough information on the distribution of SFTSV in the region. Alternatively, seroepidemiological surveys in animals can provide this information.

In this study, we attempted to identify anti-SFTSV seropositive animals by using serum samples of wild boars that were captured in Nagasaki, and we examined the infectious rates and localities of these animals.

## Methods

### Virus and cells

The YG-1 strain of SFTSV was kindly provided by Ken Maeda, Yamaguchi University. The NagH2013-1 strain of SFTSV was isolated from an SFTS patient in Nagasaki in 2013. Vero E6 cells were maintained in Eagle’s minimal essential medium (EMEM; Nissui Pharmaceutical Co.) containing 10 % fetal bovine serum (FBS). Stock SFTSV was prepared from the cell culture medium of Vero E6 cells in EMEM containing 2 % FBS. Virus titers were determined by a focus forming assay [12]. Briefly, confluent Vero E6 cells were inoculated with serially diluted culture supernatants of SFTSV and incubated in 2 % FBS EMEM containing 1 % methyl cellulose 4000 (Wako Pure Chemical Industries, Ltd.) for 5 days. Viral foci were detected by using SFTSV antiserum (source: recovered SFTS human case), peroxidase-conjugated antihuman IgG (American Qualex), and the DAB substrate (Wako Pure Chemical Industries, Ltd.). Virus titers were expressed as focus-forming units (ffu) per milliliter. The experiment using human serum was performed with the approval of the ethics committee of the Institute of Tropical Medicine, Nagasaki University (approval number: 140829129). All experiments using live SFTSV were performed in a biosafety level 3 laboratory at Nagasaki University according to standard BSL3 guidelines.

### Serum samples of wild boar

**Fig. 1:**
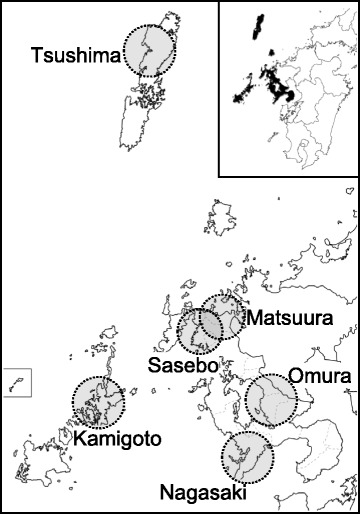
Map of the Nagasaki prefecture in the Kyushu islands, Japan. *Circles* indicate the areas where wild boars were captured. *Inset map*: Kyushu islands with the *darkened areas* indicating theNagasaki prefecture

A total of 190 serum samples were collected from wild boars that were captured in six areas of the Nagasaki prefecture (Fig. 1) from 2006 to 2012 for wild boar control conducted by Nagasaki prefecture. Samples were from juvenile (184 samples) and adult (6 samples) animals. The sera were inactivated at 56 °C for 30 min.

### Indirect IgG ELISA using recombinant SFTSV-N protein

Recombinant SFTSV-N protein was expressed and purified as previously described [13]. Recombinant Rift Valley fever virus (RVFV)-N protein was expressed and purified using the same procedure. The odd-numbered wells of rows A-G of 96-well Nunc immunoplates (Thermo Scientific, Denmark) were coated with 100 μl (50 ng/well) of recombinant SFTSV-N protein (positive antigen), and the even-numbered wells of the same rows of the plates were coated with 100 μl (50 ng/well) of RVFV-N protein (negative antigen) in PBS at pH 7.2. The plates were left at 4 °C overnight. After blocking the wells with 5 % nonfat milk (Difco, Detroit, USA) in PBS containing 0.1 % Tween 20 (PBS-T) for 1 h at 37 °C, the plates were washed three times with PBS-T. A 100-μl volume of wild boar serum diluted 1:100 with 5 % nonfat milk in PBS-T was then added to all wells previously added with either the positive or the negative antigen. Plates were incubated for 1 h at 37 °C after which they were washed as above. Then, 100 μl of 1:10,000 diluted horseradish peroxidase-conjugated goat anti-pig IgG (Bethyl Laboratories Inc., USA) was added, and incubation was done for 1 h at 37 °C. After washing the plates, 100 μl of H_2_O_2_-ABTS substrate (Kirkegaard & Perry, Gaithersburg, MD) was added to all the wells. After incubation at 37 °C for 30 min, optical density (OD) values at 410 nm were measured. Each serum sample was tested in duplicate. The adjusted OD was calculated by subtracting the OD of the well with the negative antigen from that with the positive antigen. The OD cutoff value was calculated as the mean of the adjusted OD of the negative control sera plus three times the standard deviations, generally an OD of ≤0.2 at a 1:100 sample dilution. Five negative control samples were randomly selected from negative sera determined by focus reduction neutralization test as described below. Serum samples were considered to be ELISA positive if the adjusted OD value was greater than or equal to the assay cutoff value of 0.2.

### The FRNT

Neutralizing antibody titers based on 80 % focus reduction neutralization test (FRNT) were determined for serum samples found positive by IgG ELISA. Each serum sample at different serial twofold dilutions (1:20 to 1:1280) was mixed with SFTSV (50 ffu) and incubated at 37 °C for 1 h. Each mixture of SFTSV and diluted serum sample was inoculated with duplicate in Vero E6 cells which were then incubated in 2 % FCS EMEM containing 1 % methyl cellulose 4000 for 5 days. Detection of foci of infected cells was performed as described above in the focus forming assay protocol. The neutralizing titer was determined as the reciprocal of the highest serum dilution that reduced viral foci counts by 80 %. Samples showing more than a titer of 1:20 were considered to be FRNT positive. However, we were able to observe that wild animal serum exhibited nonspecific inhibitory activity against virus infections not due to specific neutralizing antibody and few samples just below 0.2 of OD value showed low FRNT titers. Thus, in this study, we considered that samples showing both >0.2 OD value (ELISA) and >1:20 FRNT titer were seropositive for SFTSV.

## Results and discussion

**Fig. 2:**
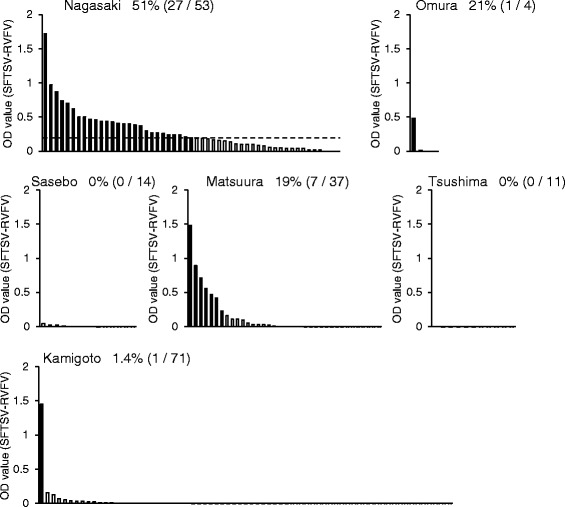
OD values after IgG ELISA of the serum samples of wild boars caught in the six areas of the Nagasaki prefecture. The *y-axis* indicates the OD values calculated by subtracting each of the OD value of the sample with the negative antigen (RVFV antigen) from the OD value of the same sample with the positive antigen (SFTSV antigen). OD cutoff value was 0.2

The serum samples of wild boars were first examined by indirect IgG ELISA for the detection of past infection with SFTSV. Wild boars with IgG seropositive samples were detected in four out of the six study areas in Nagasaki prefecture (Fig. 2). The IgG seropositive rates were 51, 25, 0, 19, 0, and 1.4 % in Nagasaki, Omura, Sasebo, Matsuura, Tsushima, and Kamigoto, respectively (Fig. 2).

We next performed an FRNT assay to confirm the positive results obtained from the IgG ELISA. All of the samples except one (K23-15) showed FRNT antibody titers of more than 1:20 (Table 1). Considering together the results of indirect IgG ELISA and FRNT, the number of SFTSV seropositive wild boars was 27 out of 53 (51 %), 1 out of 4 (25 %), and 7 out of 37 (19 %) in the areas of Nagasaki, Omura, and Matsuura, respectively. On the other hand, no seropositive wild boars were identified in Sasebo, Tsushima, and Kamigoto. There were no significant differences of positive rates between juvenile (16.7 %) and adult animals (19.0 %). Also, due to the limited number of samples, there were no significant differences in the positive rates in each area during 2006 to 2012 (data not shown). These results suggest that in the areas where SFTSV seropositive animals were found, SFTSV may be distributed in ticks and that wild boars may be frequently exposed to SFTSV.

**Table 1:** FRNT antibody titers of wild boars

Sample no.	ELISA^a^	FRNT
Nagasaki		
N21-22	1.72	1:80
N21-20	0.97	1:160
N21-15	0.87	1:160
N21-32	0.74	1:640
N21-68	0.70	1:80
N19-42	0.62	1:160
N21-8	0.50	1:320
N21-28	0.50	1:320
N21-53	0.47	1:320
N19-38	0.46	1:160
N21-17	0.44	1:40
N21-45	0.44	1:320
N19-30	0.43	1:320
N21-33	0.41	1:80
N21-6	0.40	1:80
N21-31	0.40	1:160
N21-34	0.39	1:640
N21-60	0.37	1:320
N19-26	0.30	1:160
N21-13	0.27	1:160
N21-24	0.27	1:160
N19-23	0.26	1:320
N20-3	0.24	1:160
N21-25	0.24	1:160
N21-65	0.24	1:160
N21-16	0.21	1:320
N19-39	0.20	1:160
Matsuura		
M21-4	1.48	1:160
M23-1	0.89	1:640
M24-1	0.71	1:20
M21-17	0.56	1:640
M21-11	0.47	1:160
M21-9	0.42	1:20
M22-11	0.23	1:20
Omura		
O23-1	0.48	1:160
Kamigoto		
K23-15	1.45	<1:20

^a^OD value (SFTSV-RVFV)

Although a sample of K23-15 (Kamigoto sample) showed high ELISA OD value (1.45), the FRNT antibody titer was less than 1:20 (Table 1). This raises the possibility that this animal might be infected with another virus closely related to SFTSV. Recently, new pathogenic tick-borne viruses have been reported globally, and some tick-borne viruses belonging to the genus *Phlebovirus* of the family *Bunyaviridae* have been reported [14, 15]. The earliest reported case of SFTS worldwide was from a patient in Nagasaki who was infected in 2005, suggesting that SFTSV has likely existed in this country for a long time. This raises the possibility that unknown viruses are also distributed in this area. Indeed, we isolated a new virus belonging to the genus *Nairovirus* of the family *Bunyaviridae* from ticks in Kamigoto (Shimada et al., unpublished results). Thus, further investigation of tick-borne viruses, including SFTSV and unidentified viruses in this area, will provide useful information for the development of pre-emptive countermeasures against potentially pathogenic tick-borne diseases.

In this study, we demonstrated that wild boars are likely to be useful sentinels for identifying the distributions of SFTSV. Other groups have identified SFTSV seropositive areas in Japan using animal samples from deer and dogs http://www.nih.go.jp/niid/ja/id/2244-disease-based/sa/sfts/idsc/iasr-in/4491-pr4094.html.
